# Dealing with Diversity in Digital Psychological Interventions for Young People: A Structured Review

**DOI:** 10.1055/s-0044-1788563

**Published:** 2024-07-15

**Authors:** Farzad Jahedi, Paul W. Fay Henman, Jillian C. Ryan

**Affiliations:** 1 School of Social Sciences, The University of Queensland, Brisbane, Australia; 2 Human Health, Health & Biosecurity, Commonwealth Scientific and Industrial Research Organisation, Canberra, Australia

**Keywords:** digital health, digital mental health, digital psychological intervention, demographics, social diversity

## Abstract

In recent years, despite significant progress in digital psychological interventions (DPIs), the prevalence of psychological issues among young adults remains a concern. While research on the feasibility and effectiveness of DPIs is extensive, there's a growing recognition of the need for a sociotechnical approach to enhance user engagement. This review aims to highlight the importance of integrating diversity, especially sociodemographic characteristics, into DPI design and implementation.

The review meticulously examined literature from six academic databases focused on DPIs tailored for users aged 12 to 26, spanning the period between 2009 and 2019. The data extraction process specifically targeted biosocial factors such as gender and ethnicity, as well as sociocultural elements like remoteness and labor force status among users. Among the initial pool of 879 articles, a refined selection of 25 underwent detailed analysis. Intriguingly, 14 of these studies did not treat sociodemographic factors as independent variables, leaving only 11 that did. Notably, gender and ethnicity emerged as the most frequently studied factors, with remoteness and labor force considerations receiving comparatively less attention.

Despite the acknowledged importance of user engagement in DPI effectiveness, the review highlights a critical gap: insufficient consideration of young adults' sociodemographic characteristics in intervention design and implementation. Therefore, the findings strongly support further mixed-method studies to fully understand the complex social factors influencing user engagement with DPIs. Closing this gap will undoubtedly refine and optimize DPIs to better meet the diverse needs of young adults dealing with psychological challenges.

## Background and Significance

In recent years, digital tools and technologies are poised to revolutionize mental health services, offering broad accessibility regardless of location, time, or therapist availability. 1 2 Digital psychological interventions (DPIs), digital mental health, e-mental health, or digital psychology/psychiatry all refer to the use of digital technologies (e.g., mobile phone apps, wearable sensors, virtual reality, web applications, games, etc.) designed for the prevention, diagnosis, treatment, or rehabilitation of mental health problems. 2 3 Despite the development of diverse digital solutions in the last decade, for diagnostic, therapeutic, and preventive purposes, their optimal impact hinges on addressing users' specific social needs and expectations. 2

Adolescents and young adults, facing vulnerable periods prone to mental health issues, are considered ideal DPI users due to their further involvement with digital technologies. The World Health Organization reported unipolar depressive disorders among the five most common global causes of disability in this age group in 2012. 4 Depression, a leading cause of mental illness and disability, if untreated, often persists into adulthood, incurring significant morbidity, mortality, and financial costs. 5 6 7 A body of research has argued the low usage of psychological interventions, particularly in low- and middle-income countries because of remoteness or cost of human-delivered services can be resolved by using DPIs. 8 Adolescents and young adults, adopting digital technologies earlier and spending more time online to connect, 9 emerge as ideal DPI users from a technology usage perspective.

Technology when is not adopted by users will not be effective. The analysis of technology, users, clinical adaptability, and users' social context are essential to the acceptance of a technology. 10 11 Engaging with DPIs and maintaining this engagement present significant challenges in treating mental health disorders. The high attrition rates (disengagement) during treatment with DPIs across studies revealed the impact of user engagement and psychosocial determinants of health behavior. 12 13 14 To change the current focus on the behavior change to the psychosocial determinants, Short et al 15 proposed deeper studies on both determinants and their combined effects. While engagement with DPIs can be influenced by various personal, technical, and social factors, recent studies on DPIs in young adults notably overlook the influence of social factors on engagement. 16 Recognizing young adults' diverse sociocultural characteristics is essential, as they are not a homogenous group with potentially differing needs, expectations, and attitudes. Understanding how the sociodemographical characteristics of young users influence their engagement with DPIs is crucial for achieving satisfactory mental health outcomes.

### Digital Psychological Interventions

Psychological interventions are nonpharmacological solutions to assist people to develop skills needed for tackling risks, mediators, or bouts of mental illnesses and empower patients in social situations. 17 These interventions are based on cognitive, behavioral, and interpersonal theories to provide patients with psychosocial rather than pharmacological therapies. DPIs leverage digital solutions to monitor, prevent, treat, and support individuals with mental health disorders. 17 In societies where electronic engagement is prevalent, DPIs prove particularly beneficial. 17 Young adults, accustomed to pervasive digital activities, because of the omnipresence of digital activities in their lives. 18 However, research highlights challenges in DPI use related to design, usability, privacy, data protection, and cost. 19 Bhugra et al's 2 comprehensive review underscores the impact of patient and clinician engagement, clinical validation, interoperability, scalability, and economic value on DPI potential. While digital solutions enable widespread treatment delivery, personalization of DPIs is crucial. Understanding individual and social perspectives on DPI design and implementation poses challenges, requiring designers and clinicians to delve deeply into these dimensions.

### Mental Health and Social Demographics

Health sociology has extensively explored health's social and cultural dimensions, acknowledging gender, ethnicity, income, and education as crucial sociodemographic health determinants. Additionally, social considerations come into play when evaluating responses to digital technology impacts. 20 21 Various social characteristics, such as socioeconomic status, sex/gender, age, ethnicity, and social roles like marriage, parenting, or employment, 22 or geographical context, which can be characterized as urban, rural, and remote 23 interact with psychological disorders. Sociodemographic factors encompass biosocial traits (age, gender, ethnicity) and sociocultural traits are based on an individual's position or status in society (marital status, income, occupation, education, religion). 24 Recognizing the significance of sociodemographic characteristics in mental health experiences is essential in studying DPIs. A deeper understanding of how these characteristics influence DPI engagement enables better responses to diverse client experiences through appropriately designed interventions.

“Gender” encompasses social and cultural characteristics associated with biological sex, involving gender identity and stereotypes (beliefs about behaviors, dress, interactions, societal positions, etc.). The internal sense of gender is called gender identity while the categorization of male and female is based on the gender stereotypes of their gender performance. 25 “Ethnicity,” distinct from race, connects people based on shared culture and background, involving cultural differences and attitudes to kinship. 25 26 Ethnicity is self-identified, shaped by cultural and nationality psychosocial characteristics (such as language, religion, and history). 27 While ethnicity is identified by individuals, its boundaries are not defined by countries but by groups of people and how the members of these groups interact internally and externally. 28 “Remoteness,” defined by proximity to urban and rural areas, varies based on geographic and sociodemographic factors. 29 “Labor force” or work status indicates user employment/unemployment status. 24 Understanding these sociodemographic dimensions is crucial for effective DPI design and implementation, enabling tailored interventions that consider diverse client experiences.

### Previous Reviews

Most relevant and recent (published 2019–2021) reviews on digital mental health or digital psychology can be classified into four different research focuses. The notable focuses of these studies have been the clinical and preventive effectiveness of DPIs 30 31 32 33 34 as well as their design. 35 36 37 38 However, the social 39 40 and economic 41 42 aspects of DPIs have not received much attention. Although these reviews focused on various aspects of DPIs, the agreed challenge is engaging with and retaining users. Even the most clinically effective and cost-effective DPIs will not help when their design discourages engagement. A combination of technical, psychological, and social approaches to the design, implementation, and study of DPIs provides designers, clinicians, and users with valuable information for improving and promoting engaging DPIs. These gaps in the understanding of how sociodemographic diversity influences young users' engagement with DPIs were the motivations for this review to understand how sociodemographic (biosocial and sociocultural) characteristics, of young users, are considered in the design and implementation studies of DPIs.

## Methods

**Fig. 1:**
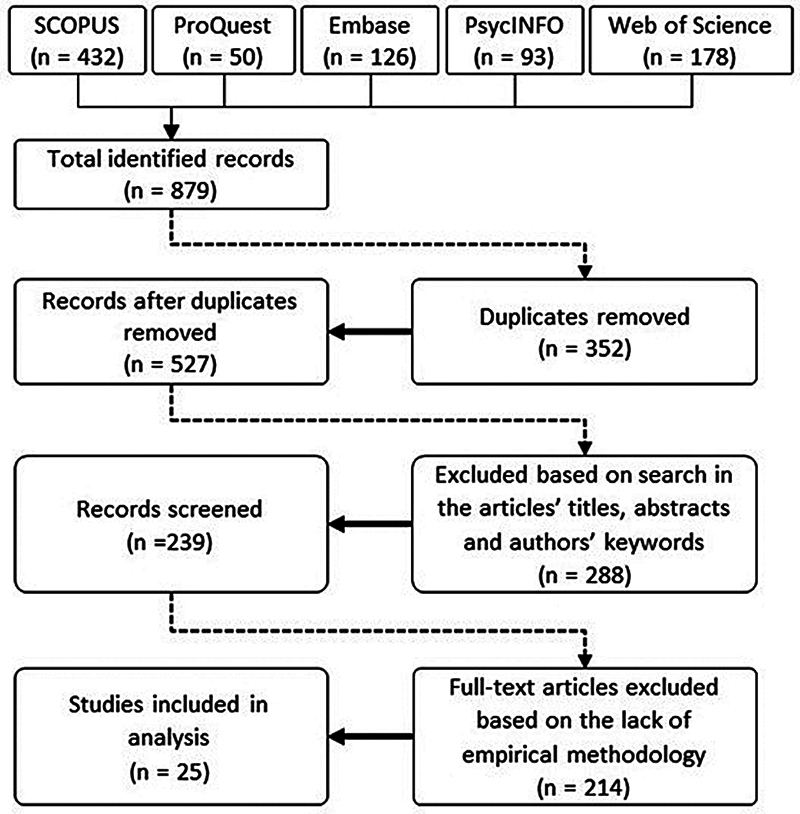
Modified PRISMA-based steps. 44 PRISMA, Preferred Reporting Items for Systematic reviews and Meta-Analyses.

This systematized review 43 is a structured review similar to a systematic review; however, some elements of the guidelines are relaxed. With the same approach, this review is designed based on some elements of PRISMA (Preferred Reporting Items for Systematic reviews and Meta-Analyses) guidelines ( Fig. 1 ). 44 Screening articles and data extraction were conducted by one reviewer who consulted regularly with the broader authorship team. Like a scoping review, the quality appraisal was not undertaken.

### Search Strategy

The search strategy was designed in consultation with an academic librarian at the University of Queensland (Australia). In a preliminary scoping search, two major concepts (“mental health” and “digital”) and their synonyms were defined and combined by Boolean operators as a generic search phrase ( Table 1 ). The generic search phrases were modified and applied for each target journal database (SCOPUS, ProQuest, ScienceDirect, Embase, PsycINFO, Web of Science). The modifications made for each database were informed by varying formats of the search phrase composition in conjunction with filters, resulting in ScienceDirect yielding no results. The search phrase targeted to find the keywords anywhere in the title, keywords, and abstracts of the papers. In the first step, publication date (2009–2019), document type (peer-reviewed articles), and language (English) criteria were applied, and the targeted age group of adolescents and young adults (12–26 years old) was filtered to refine the search results. The search was conducted on titles, keywords, and abstracts of the publications. Searches identified 879 articles including 352 duplicates, which lead us to screen 527 articles ( Fig. 1 ).

**Table 1:** The search strategy, keywords, search phrase

Search keywords	
Mental health	Digital
• Mental health • Mental disorders • Psychological disorders • Mental health intervention • Psychological intervention	• Digital • Electronic • Computer • Internet • Mobile • Virtual
**Generic search phrase**	
(“mental health intervention” OR “psychological intervention”) AND (“digital” OR “mobile” OR “internet” OR “mobile” OR “electronic” OR “virtual”)	

### Study Selection and Inclusion Criteria

The screening of the mentioned 527 articles resulted in the discovery of 239 relevant articles. This screening process was conducted using NVivo (ver. 12.0), searching for keywords in the titles, abstracts, and authors' keywords of the articles. The 288 excluded articles did not address digital mental health interventions or related keywords. In the next step, the full-text articles were reviewed for relevancy based on their methodology, study focus, and design. Papers without an empirical study of the digital intervention for the treatment of the psychological issue were excluded such as systematic or nonsystematic reviews, prototype design, theoretical papers, or surveys about the DPIs without any exposure of the participants to the intervention (214 articles), resulting in 25 articles for data analysis.

### Data Extraction and Synthesis

General and specific data were extracted from the selected 25 studies using a taxonomy with hierarchical attributes ( Table 2 ). Classification of how papers dealt with diversity or the sociodemographic characteristics of participants in their design or implementation of DPIs was the focus of our review. We tried to understand how the researchers approached gender, ethnicity, remoteness, and labor force status of users (participants) in a specific DPI design or during the implementation or trial of the DPI among these different sociodemographic-based subpopulations. The methodology, results, and discussion sections of papers were scrutinized to extract the sociodemographic data considered as variables either in the implementation of DPIs or as the centerpiece in the design of the sociodemographic-tailored intervention. Moreover, the psychological diagnoses of participants, the type of intervention, and general study characteristics (focus, method, and location) were collected. These extractions were based on the following definitions and conducted by the first author, whereas doubtful cases were decided by the authorship team.

**Table 2:** Taxonomy of the key concepts used for coding and analysis

1. Participant sociodemographic characteristics 1.1. Biosocial 1.1.1. Age 1.1.2. Gender 1.1.3. Ethnicity 1.2. Sociocultural 1.2.1. Remoteness (urban vs. rural) 1.2.2. Labor force status 2. Psychological factors 2.1. Mental health disorder by DSM-5 2.2. Psychological intervention 2.2.1. Type 2.2.2. Clinical focus 3. Study design 3.1. Research study design (method) 3.2. Research focus 3.3. Country of the research (location)

Abbreviation: DSM-5, fifth edition of the Diagnostic and Statistical Manual of Mental Disorders.

The key concepts of taxonomy were defined by literature and traced in the papers. Demography and its categories were classified into biosocial (gender and ethnicity) and sociocultural (remoteness and labor force) traits. 24 The “Gender” data was extracted based on how the researchers considered or approached the gender-specific design of the DPIs or implementation in gender-based subpopulations. For “Ethnicity,” any ethnicity-specific design or implementation of DPIs for a specific ethnicity subpopulation in the papers was identified. While researchers were aware of race and ethnicity differences, this review did not differentiate them in the data synthesis to maintain the simplicity and consistency. Regarding “Remoteness,” papers were investigated of DPIs that specifically designed for remote areas inhabitants or compared the implementation in different users' living places. The “Labor force” was based on whether work status (employment/unemployment) was considered in the design or implementation of DPIs. These sociodemographic data were extracted where the participants' genders, ethnicities, living places, and labor force status were considered independent variables or critical factors in the design or implementation of the DPIs. Psychological attributes were defined as “mental health disorders” following the fifth edition of the Diagnostic and Statistical Manual of Mental Disorders 45 and types of “psychological interventions” based on the Australian Psychology Association's Classification. 46 The study design also focused on the method and focus of the study. This classification reached the current maturity by amendments during the analysis process to cover these terms. Zotero, 47 NVivo (ver. 12.0) and MS Excel were used to manage data collection and arranging of the resources, coding, and classification.

## Results

### Study Characteristics

The 25 articles' overall findings are presented grouped by their focus, methods, and countries. Studies were categorized based on assessing clinical effectiveness, technological acceptance, feasibility, understanding users' perspectives, and cost-effectiveness of DPIs ( Table 3 ).

**Table 3:** The overall description and findings of the articles reviewed in this study

Article's title	Brief description	Results
A little effort can withstand the hardship: fielding an internet-based intervention to prevent depression among urban racial/ethnic minority adolescents in a primary care setting 85	• Mixed methods • Assess depression screening • Internet-based mental health interventions • For urban minority adolescents	• Varied screening success rates • Identified community barriers to implementation
A mobile phone application for the assessment and management of youth mental health problems in primary care: a randomised controlled trial 79	• Examining the effectiveness of • “Mobiletype” a mental health assessment and management mobile application • In primary care settings	• Significantly increased Emotional Self Awareness (ESA) • It did not have significant effects on depression, anxiety, or stress
A mobile phone-based intervention to improve mental health among homeless young adults: pilot feasibility trial 54	• Develop and test the feasibility and acceptability of • A remotely delivered mental health intervention • For young adults experiencing homelessness	• High rates of satisfaction with the intervention • Minimal changes in clinical outcomes • Need for further exploration
A virtual reality–based psychological treatment in long-term hospitalization: a case study 73	• A psychological intervention for • Children and young patients • Using virtual reality videogames and telepsychology • To improve resilience and coping strategies during long-term hospitalization	• Treatment's feasibility and efficacy in reducing anxiety and enhancing emotional competence • The potential of technology-based interventions in clinical psychology
A qualitative study of sexual minority young people's experiences of computerised therapy for depression 87	• Experiences of • Sexual minority youth • Using computerized therapy for depression, • Revealing themes of appeal and areas for improvement	• Incorporating more sexuality-specific content • Found computerized therapy helpful in reducing depression • Its potential for underserved groups
Access and completion of a web-based treatment in a population-based sample of tornado-affected adolescents 48	• Assessed adolescents' access and completion rates of • A Web-based treatment • For postdisaster mental health symptoms	• An overall access rate of 35.8% and varying completion rates • Parental internet use was associated with increased access • Adolescent males were less likely to access the treatment • The need for strategies to enhance the reach of such treatments in postdisaster contexts
Indigenous adolescents' perception of an eMental Health Program (SPARX): exploratory qualitative assessment 53	• Māori adolescents' opinions • On the SPARX program	• Its cultural designs facilitated engagement • Led to improved mood and hope
Internet-based cognitive behavioral therapy for adolescents with anxiety disorders: a feasibility study 86	• Feasibility of a translated and adapted • Internet-based CBT program • For adolescents • With anxiety disorders • In Denmark	• The program is generally well-received • Promising clinical improvements, • Its potential as a psychological intervention for Danish adolescents
Longitudinal change in parent and child functioning after internet-delivered cognitive-behavioral therapy for chronic pain 69	• Longitudinal associations between • Parent and child functioning in youth with chronic pain • Receiving family-based digital cognitive-behavioral therapy	• Higher parent distress at pretreatment may hinder improvement in child disability over time, • The need to consider and target parent distress in interventions for pediatric chronic pain
Rural and urban/suburban families' use of a web-based mental health intervention 83	• Access and completion of • A Web-based mental health intervention • Among rural and urban/suburban adolescents • And their caregivers • Affected by tornadoes	• No differences in access or completion rates based on geographical location, • Potential for Web-based resources to be utilized across different communities
The effectiveness of therapeutic play, using virtual reality computer games, in promoting the psychological well-being of children hospitalised with cancer 82	• Assess the effectiveness of • Therapeutic play, using virtual reality computer games, • In reducing depressive symptoms • In Hong Kong Chinese children hospitalized with cancer	• Significant reduction in depressive symptoms • The potential of therapeutic play interventions in promoting holistic care for hospitalized children
Evaluation of the effectiveness of a musical cognitive restructuring app for Black inner-city girls: survey, usage, and focus group evaluation 50	• Assess the effectiveness of • The Build Your Own Theme Song (BYOTS) app • In reducing negative thinking and anxiety • Among Black adolescent girls	• Significant reduction in negative thinking and anxiety • The app's potential as a useful tool in delivering cognitive restructuring in an underserved urban population
Web intervention for adolescents affected by disaster: population-based randomized controlled trial 70	• Evaluate the effectiveness of • Bounce Back Now (BBN), a web-based intervention, • For disaster-affected • Adolescents and parents	• Fewer symptoms of posttraumatic stress disorder and depression among adolescents in the BBN group • The feasibility and initial efficacy of BBN as a scalable disaster mental health intervention
Impact of an acceptance facilitating intervention on patients' acceptance of internet-based pain interventions: a randomized controlled trial 84	• Evaluate the effectiveness of • An acceptance-facilitating • Internet-based psychological pain interventions	• Significantly high acceptance levels • The potential of such interventions to positively influence patients' engagement
Computer-assisted cognitive remediation therapy: cognition, self-esteem and quality of life in schizophrenia 75	• Investigate the effects of • Computer-assisted cognitive remediation therapy (CACR) • On schizophrenia patients' neurocognitive outcomes, quality of life, and self-esteem	• Improvements in neuropsychological performance, quality of life, and self-esteem • The potential benefits of this intervention for individuals with schizophrenia
Cross-sectional survey evaluating Text4Mood: mobile health program to reduce psychological treatment gap in mental healthcare in Alberta through daily supportive text messages 71	• Assessed the impact of • Delivering daily supportive text messages by Text4Mood • On subscribers' mental wellbeing	• High acceptance and effectiveness • Supportive text messages offer a feasible and beneficial adjunctive intervention for individuals with mental health concerns
Effects of a cognitive behavioral self-help program on depressed mood for people with acquired chronic physical impairments: a pilot randomized controlled trial 76	• Assess the effectiveness of • A cognitive-behavioral self-help intervention • For improving depressed mood • In individuals with chronic physical impairments	• Significant improvements in depression scores post-intervention and at a 2-month follow-up • The potential of self-help programs as cost-effective and accessible interventions
Randomized controlled pilot trial of supportive text messages for patients with depression 55	• Assess the efficacy of • Supportive text messaging • In improving treatment outcomes • For depressed patients	• Significant improvements in depression scores and self-rated health • Their potential as a psychological intervention for depression, particularly in underserved populations
Initial open trial of a computerized behavioral activation treatment for depression 72	• An open trial suggested that • The “Building a Meaningful Life Through Behavioral Activation” • Led to significant improvements in depression scores • For moderately to severely depressed individuals	• Further investigation into the program's effectiveness
Internet-based cognitive therapy for social anxiety disorder in Hong Kong: therapist training and dissemination case series 81	• Therapist training for • Delivering internet-based cognitive therapy • For social anxiety disorder (iCT-SAD)	• Effective in increasing therapists' knowledge and skills • With patients achieving significant improvements •iCT-SAD is feasible, acceptable, and shows promising initial efficacy in the Hong Kong cultural context, • Warranting further investigation through an RCT
Internet-delivered treatment for young adults with anxiety and depression: evaluation in routine clinical care and comparison with research trial outcomes 80	• Acceptance and effectiveness of • An internet-delivered psychological intervention (Mood Mechanic) • For depression and anxiety symptoms • In young adults	• Effective and well-received both in a research trial and routine healthcare setting • Significant symptom reductions • High treatment satisfaction reported • Absence of a control group • Lack of formal diagnosis
The Second Randomised Evaluation of the Effectiveness, Cost-Effectiveness and Acceptability of Computerised Therapy (REEACT-2) trial: does the provision of telephone support enhance the effectiveness of computer-delivered cognitive behavior therapy? A randomized controlled trial 78	• Evaluation of the effectiveness, cost-effectiveness and acceptability of • Telephone-facilitated free-to-use computerized cognitive behavior therapy (cCBT) • A randomized controlled trial	• Short-term benefits with lower depression severity • Higher odds of no longer being depressed at 4 months • Not significant at 12 months. • Small to moderate effects that should be considered when offering cCBT for depression
The clinical effectiveness of web-based cognitive behavioral therapy with face-to-face therapist support for depressed primary care patients: randomized controlled trial 77	• Clinical effectiveness study of • Guided Web-based intervention (combined MoodGYM and therapist support) • In reducing depression and anxiety symptoms • In primary care patients	• Effective in reducing depression and anxiety symptoms • Moderate rates of nonadherence • Overall treatment satisfaction was high • Positive gains largely maintained at 6-mo follow-up
Time for a change: college students' preference for technology-mediated versus face-to-face help for emotional distress 49	• Preference study of • College students • Being open to seek emotional help • Through online therapy and/or serious games versus face-to-face	• A potential for online evidence-based treatments. • Ethnicity and level of emotional distress did not significantly affect help-seeking preferences among participants
Uptake and adherence of a self-directed internet-based mental health intervention with tailored e-mail reminders in senior high schools in Norway 74	• Adherence study of • A self-directed internet-based mental health intervention • In high schools faced challenges	• Low uptake and adherence rates despite tailored email reminders • Effective communication about the program's usefulness is essential

In terms of methods ( Table 4 ), the prevalent approach was randomized controlled trials (RCT), complemented by other quantitative methods such as pre–post design and case–control. Additionally, several studies incorporated qualitative or mixed methods in their research design.

**Table 4:** General characteristics of the review (focuses and methods)

Focus of the studies	*N*	References
Assessing the clinical effectiveness of a DPI	17	50 54 55 69 70 71 72 73 74 75 76 77 78 79 80 81 82
Technological acceptance	7	48 49 54 77 81 83 84
Feasibility of DPIs	5	54 74 81 85 86
Understanding users' perspectives of the DPI	3	53 71 87
Cost-effectiveness	1	78
Methods of the studies	*N*	References
RCT	10	55 69 70 74 75 76 77 78 79 85
Pre–post design	5	48 50 54 72 86
Case–control	4	48 82 83 84
Cohort (retro/pro)	1	80
Cross-section	1	49
Case study	2	73 81
Qualitative or mixed methods	7	48 50 53 71 72 86 87

Abbreviations: DPI, digital psychological intervention; RCT, randomized controlled trial.

### Psychological Conditions and Interventions

DPI studies predominantly centered around depressive disorders, with anxiety disorders and trauma- and stressor-related disorders following closely ( Table 5 ). While many articles explored multiple disorders, others fell into three classified groups: substance use and addictive disorders, somatic symptom disorders, and schizophrenia spectrum and other psychotic disorders. Examining the types of psychological intervention (see the second part of Table 5 ), cognitive behavioral therapy was overwhelmingly employed in DPIs (76%), followed by behavioral activation (20%), psychoeducation (16%), and interpersonal therapy (12%). The third part of Table 4 indicates that, in many instances, DPIs addressed multiple clinical focuses, with the treatment of psychological issues being the primary goal, followed by prevention. While DPIs were utilized for monitoring (8%) and rehabilitation (4%) of psychological issues, their application for diagnosis was not evident.

**Table 5:** Psychological disorders, implemented psychological interventions, and clinical focus in the digital psychological interventions

Psychological disorders (based on DSM-5)	*N*	References
Depressive disorders	19	48 49 53 54 55 70 71 72 73 74 76 77 78 79 82 83 85 87
Anxiety disorders	9	49 50 71 73 79 80 81 82
Trauma- and stressor-related disorders	5	48 54 70 71 83
Substance use and addictive disorders	3	70 71 83
Schizophrenia spectrum and other psychotic disorders	2	71 75
Somatic symptom disorders	2	69 84
Bipolar and related disorders	1	71
Personality disorders	1	71
Other disorders	1	71
Psychological interventions (based on APS)	*N*	References
Cognitive behavior therapy (CBT)	19	48 49 50 53 54 55 69 70 71 74 76 77 78 80 81 83 85 86 87
Behavioral activation	5	48 70 72 83 85
Psychoeducation	4	48 70 83 86
Interpersonal psychotherapy (IPT)	3	49 74 85
Mental health assessment and management (monitoring)	2	54 79
Acceptance and commitment therapy (ACT)	1	73
Community resiliency concept model (emotion management)	1	85
Dialectical behavior therapy (DBT)	1	73
Play therapy (children)	1	82
Positive affect and hedonic and eudemonic well-being	1	73
Cognitive remediation therapy/rehabilitation	1	75
Psychological pain interventions (based on CBT)	1	84
Clinical focus	*N*	References
Treatment	21	48 49 50 53 54 55 69 70 71 72 73 74 76 77 78 80 81 82 84 86 87
Prevention	5	49 71 74 83 85
Monitoring	2	54 79
Rehabilitation	1	75
Diagnosis	0	–

Abbreviations: APS, Australian Psychology Association; DSM-5, fifth edition of the Diagnostic and Statistical Manual of Mental Disorders.

### Sociodemographic Characteristics in the Digital Psychological Intervention Studies

In examining how studies considered users' sociodemographic characteristics, we differentiated between those that included these characteristics in their participant profiles (referred to as “reported”) and those that analyzed and reported findings based on these characteristics (referred to as “studied”). Within our selected papers, participants' gender was reported in 16 papers, but actually studied only in 9 ( Table 6 ). This ratio (reported to studied) is less about ethnicity (11:4), remoteness (11:2), and labor force (9:2). To comprehend the correlation of sociodemographic characteristics with DPIs, it is crucial to study these characteristics as a research variable rather than merely reporting them to describe the participants. Our review depicted low rate of these sociodemographic studies: gender (36%), ethnicity (16%), remoteness (8%), and labor force status (8%). Therefore, the “reported only” row in Table 6 indicates the indirect incorporation of sociodemographic characteristics into studies (on average, approximately half of the time), where the scientific deductions about these characteristics are shallow.

**Table 6:** Sociodemographic characteristics in the reviewed publications

	Gender (%)		Ethnicity (%)		Remoteness (%)		Labor force (%)	
Not reported	0	(0)	10	(40)	12	(48)	14	(56)
Reported only	16	(64)	11	(44)	11	(44)	9	(36)
Studied	9	(36)	4	(16)	2	(8)	2	(8)
	25		25		25		25	

#### Gender

Nine papers that studied gender in the DPIs can be categorized into two groups. The first group examined the clinical effectiveness and acceptance of DPIs among different gender groups. For instance, Price et al 48 found boys were more likely to initiate engagement with the DPI than girls and Lungu and Sun 49 found women had a greater propensity to access online therapy than men. The other group designed gender-oriented DPIs. It appeared the target groups perceived these designs as engaging. For example, a design based on girls' favorite song themes was not only interesting to the girls, but also their mothers expressed interest in using the DPI. 50 Although generally, the gender-oriented design of interface in digital solutions showed significant satisfaction of the target gender, 51 our review revealed mixed results about gender-based differences in the effectiveness, acceptance, and design of the DPIs.

#### Ethnicity

Ethnicity was not examined in 84% of our reviewed studies, which suggests the ethnicity of young people has not been well recognized by the researchers in DPI studies over the last decade. Among those four studies considering ethnicity, one group focused on the single ethnic-related design effectiveness or users' satisfaction, whereas the other group compared the engagement or satisfaction of participants with different ethnicities on the implemented DPIs. All in all, these research suggested ethnicity-oriented designs improve the low uptake of DPIs 52 and gain more appreciation from the target subpopulation. 50 53

#### Remoteness

Remoteness was reported in 14 studies by mentioning the location of the study or the living place of participants. However, only two analyzed this characteristic as a variable ( Table 4 ). Both studies focused on the participants' attitudes toward DPI in urban versus rural or specific disaster areas in the United States, but they did not find any significant differences.

#### Labor Force Status

Our review revealed the labor force or employment status is overlooked in the DPI studies, with only two papers examining it empirically as a variable ( Table 4 ). These two studies found no significant correlation between DPI use or effectiveness by labor force status. One qualitative study in a small group found no difference in participants' satisfaction among employed, unemployed, and student groups. 54 In the other one, an RCT study found employment uninfluential on the efficacy of depression treatment. 55

## Discussion

This study reviewed 25 papers considering sociodemographic diversity in the design and implementation of DPIs. DPIs mostly focused on depressive and anxiety disorders and CBT was the most common approach underpinning the DPI. The majority of reviewed studies did not empirically evaluate the sociodemographic characteristics of participants in association with DPI. Notably, only 8 to 36% ( Table 4 ) of papers analyzed these characteristics as variables. Gender was the most frequently reported and studied sociodemographic characteristic, whereas labor force status was studied the least. Of those that did, the biosocial characteristics (gender and ethnicity) were the most common. While these characteristics are widely used in health research, 56 our review's findings implied these sociodemographic aspects of DPIs are not yet considered empirically in the analysis, design, or implementation of DPIs. This review observed two different ways in which the reviewed studies considered DPI users' sociodemographic characteristics: considering the users' characteristics in design where some studies designed DPIs specifically to take account of such characteristics; investigating how such characteristics affect the implementation of DPIs and assessed the impact of these characteristics on the use and outcome of DPIs. These findings are discussed in more detail in the sections below.

### Sociodemographic in Design

Design based on the users' needs and preferences is the key to more successful, interactive, and user-centered digital solutions. Ethnographic research has been used by designers since 1970 to learn more about users. 57 However, our review of a decade of studies shows that sociodemographic characteristics of young adults are rarely at the center of DPI design. We investigated four characteristics (gender, ethnicity, remoteness, and labor force status) in these studies. The gender-oriented designed DPIs were perceived as engaging by the target groups. For example, a design based on girls' favorite song themes was not only interesting to the girls, but also to their mothers. 50 Ethnicity-oriented design is suggested by Kayrouz et al 52 to improve the low uptake of DPIs, and the designs to reflect the ethnicity gained more appreciation from the target subpopulation. 50 53 In line with this approach, culture, as an important structure of societies, has been studied from different perspectives to provide digital technology designers with standards or models for developing more usable and engaging interfaces. 58 59 60 Although users' ethnicity is found associated with the perception of the usefulness of DPI and engagement among the users, 61 overlooking the ethnicity in the design of DPIs for young people is our finding that needs future ethnicity-oriented studies and designs among young adults to enhance our knowledge on how ethnicity may mediate DPI engagement and efficacy. Remoteness and labor force status were not considered in any DPI designs reviewed.

### Sociodemographic in Implementation

Among a broad range of frameworks, individual users have been one of the main focuses of implementation science, 62 in which as a process, the context of implementation is interlaced with the social nature of users. 63 Implementation of digital health interventions became a scientific domain to improve the quality and effectiveness of health services and avoid failures. 64 We found the studies that evaluated the implementation of DPIs did not report any gender-based differences in clinical effectiveness, but gender differences were found in the acceptance of DPI. For instance, Price et al 48 found boys were more likely to initiate engagement with the DPI than girls or Lungu and Sun 49 found women had a greater propensity to access online therapy than men. Although generally, the gender-oriented design of interface in digital solutions showed significant satisfaction of the target gender, 51 our review revealed mixed results about gender-based differences but in the effectiveness, acceptance, and design of the DPIs. These mixed results might be because of the quantitative methods in these papers that focused on the differences in design, DPI purpose, and so on. Future qualitative or mixed methods can help to provide more understanding and clarification.

Among those studies that considered ethnicity, only one study compared the engagement or satisfaction of participants of different ethnicities with the implemented DPIs. We also investigated the sociocultural characteristics of living location and labor force status. 24 Two studies considered “living locations” and compared the participants' attitudes toward DPI in urban versus rural or disaster areas in the United States but did not find any significant differences. This nondifference may not be evident, if the studies were undertaken in other locations. Underlying digital exclusion in countries at similar developmental levels might be the same. For instance, Park's 65 study on remoteness in Australia as an indicator of Internet connectivity did not reveal a significant difference. Moreover, the mentioned result may reflect the high penetration of DPIs among youth in both regions, while still a geographical digital divide in access to appropriate digital infrastructure may impact the users. Furthermore, regardless of the access issues, other social complex characteristics and values may cause differences. These variances may impact the users' engagement with the DPI differently in the urban and remote areas, warranting further research.

“Labor force” or employment status is mostly overlooked in the DPI studies. Two studies found no significant correlation between DPI and the labor force status; Schueller et al's 54 qualitative study of users' satisfaction found no difference among employed and unemployed groups, and an RCT study found employment uninfluential on the efficacy of depression treatment. 55 These findings might be due to their superficial approach to the employment status of participants while the labor force is a complex sociodemographic variable interacting with multiple social, economic, and personal factors. Thus, in-depth studies are needed to further the understanding of this characteristic in the users' engagement with DPIs.

Looking back to our results indicates the real need for a sociotechnical approach for more complex design and implementation of DPIs for young adults. The design and implementation of DPIs need to merge technical, psychological, and social approaches to make the product or the service more effective, feasible, and engaging. Even though codesign is capable to produce engaging digital solutions for young people, 35 it is uncommon for DPIs. 30 DPI design and implementation have social, psychological, and technical aspects that substantiate the importance of the user experience and integrating the solution with communities, caregivers, patients, and health practitioners, as the successful approaches. 66 Despite the knowledge about the role of sociodemographic factors in the perception of participants, which enhance their engagement, many DPI studies either have disregarded these factors or have superficially investigated these factors in their design or implementation. Moreover, a few studies that evaluate the users' acceptance of the DPIs need more complex studies to scrutinize the quality of their perception and the influencing characteristics on the longevity of their usage or engagement.

While recent reviews have delved into various aspects of engagement with DPIs from the perspective of treatment burden, providing less focus on sociodemographic factors, 67 a review of literature spanning from 2007 to 2022 corroborated our findings by revealing a prevalent lack of reporting on these factors in RCT studies of DPIs. 68 The primary revelation of our review underscored the deficiency in directly studying sociodemographic characteristics as primary variables in the analysis of DPIs. The practical implication of this observation conveys a crucial message to DPI designers and clinicians. Designers should involve users in the design process to incorporate their sociodemographic characteristics in content preparation, interface design, and interactions. Clinicians should take into account patients' sociodemographic profiles when prescribing DPIs or formulating treatment plans for individuals of varying ages, genders, ethnicities, labor force statuses, or levels of remoteness.

## Strengths and Limitations

Our review exhibited strengths in employing a systematic search approach, establishing a straightforward taxonomy for data extraction, and benefiting from a collaborative effort involving researchers from diverse fields such as psychology, sociology, and digital health. It builds upon prior reviews focused on the sociology of DPIs for adolescents and young adults. However, certain limitations should be acknowledged. There is a possibility that other relevant articles, either published after 2019 or available in different journal databases, were not included in this research. Additionally, the inclusion and exclusion process lacked blind review by multiple researchers. While one researcher managed all search and content examination steps, critical decisions were made collectively by the team of researchers who remained updated on the details.

## Conclusion

In conclusion, this review scrutinized sociodemographic characteristics in DPI studies focusing on adolescents and young adults over a decade (2009–2019). The findings reveal a significant neglect of sociodemographic factors—specifically, gender, ethnicity, remoteness, and labor force status—in the design and implementation of DPIs for young adults. While approximately half of the reviewed studies reported participant sociodemographic characteristics, only a third incorporated these characteristics in their research. Gender emerged as the most studied sociodemographic characteristic (53%), followed by ethnicity (23.5%), showcasing diverse impacts on DPI implementation.

This suggests a potential oversight in the analytical examination of social determinants concerning digital health among young people. Future research should emphasize the exploration and evaluation of DPI design and implementation, incorporating diversity, social factors, and considering psychological and technological perspectives. Introducing flexibility through personalized options for users and collaborative codesign of DPIs, accounting for sociodemographic diversity, can enhance user satisfaction and acceptance. Despite numerous successful feasibility and clinical effectiveness studies, further comprehensive qualitative and quantitative exploration of biosocial and sociocultural characteristics, such as ethnicity, remoteness, and labor force, is recommended.

## Clinical Relevance Statement

This study emphasized the importance of incorporating sociodemographic factors in the design, evaluation, and empirical research of DPIs. It identified a conspicuous gap in the examination of sociodemographic characteristics within studies of DPIs targeting young adults. The implications of this study extend to guiding clinicians, designers, and policymakers in addressing psychological interventions for young adults, as well as directing researchers' attention toward empirical sociodemographic studies related to DPIs.
